# Circadian rhythm and aggressive behavior in community-dwelling schizophrenia patients testing a moderated mediation model

**DOI:** 10.3389/fpsyt.2025.1741109

**Published:** 2026-01-14

**Authors:** Li-Juan Duan, Dong-Mei Wu, Meng-Lin Jiang, Yu-Tao Wang, Zi-Yi Duan, Jia-Lu Xu, Rui-Tong Miao, Wei-Dong Lin, Yan Jiang

**Affiliations:** 1 West China School of Nursing, Sichuan University, Chengdu, China; 2 Department of Neurosurgery, West China Hospital, Sichuan University, Chengdu, China; 3 Department of Nursing, West China Xiamen Hospital of Sichuan University, Xiamen, China; 4 Department of Nursing, The Clinical Hospital of Chengdu Brain Science Institute, Ministry of Education (MOE) Key Laboratory for Neuroinformation, University of Electronic Science and Technology of China, Chengdu, China; 5 Center for Neuropsychiatric Disorders, West China Xiamen Hospital of Sichuan University, Xiamen, China; 6 School of Laboratory Medicine, Chengdu Medical College, Chengdu, China; 7 Department of Nursing, West China Hospital, Sichuan University, Chengdu, China

**Keywords:** aggressive behavior, circadian rhythm, perceived social support, schizophrenia, sleep disturbance

## Abstract

**Background:**

Aggressive behavior in patients with schizophrenia poses significant challenges to community management. Although disruption of circadian rhythm is a recognized risk factor, the underlying mechanisms related to sleep disorders and social support remain unclear.

**Objective:**

This study conducted a cross-sectional survey to investigate the impact of circadian rhythm on aggressive behavior in community-dwelling schizophrenia patients, and tested a moderate mediation model focusing on sleep disorders and perceived social support.

**Method:**

A total of 818 patients with schizophrenia were recruited from 28 communities in China. After data quality checks, 785 participants (effective response rate: 95.97%) were included in the final analysis. Participants completed self-report measures assessing chronotype (Morningness-Eveningness Questionnaire-5, MEQ-5), sleep quality (Pittsburgh Sleep Quality Index, PSQI), perceived social support (Perceived Social Support Scale, PSSS), and aggressive behavior over the past month (Modified Overt Aggression Scale, MOAS). A moderated mediation model was tested using the PROCESS macro.

**Results:**

The results demonstrated that circadian rhythm(as assessed by chronotype preference) are associated with aggressive behavior both directly and indirectly through sleep disorders. Furthermore, perceived social support moderated the relationships among circadian rhythm, sleep disorders, and aggressive behavior by providing a tripartite protective function.

**Conclusion:**

In this clinically stable community sample, eveningness preference and sleep disturbance were associated with higher levels of self-reported aggression, while perceived social support appeared to buffer these relationships. These cross-sectional findings suggest potential targets for psychosocial intervention but require longitudinal confirmation.

## Introduction

Schizophrenia, a severe mental disorder with an incompletely understood etiology, is characterized by significant multidimensional functional impairment. This impairment manifests as social and occupational dysfunction, high relapse rates, and chronic disability, imposing a substantial burden on patients’ families and society. Globally, approximately 25 million people are affected by schizophrenia (1). While most patients transition to community rehabilitation after achieving symptom stability post-hospitalization, their quality of life typically remains low. Furthermore, approximately one-third of these patients (with a total prevalence rate of 33.3%) exhibited aggressive behavior (2). This factor largely contributed to the stigmatization associated with schizophrenia and indicated a significantly increased risk of violent behavior. Specifically, aggressive behavior in these patients—defined as destructive actions against oneself, others, or property, often triggered by psychiatric symptoms or environmental factors—occurs at rates 2–10 times higher than in the general population (3). This behavior poses public safety risks and subjects patients to severe social stigma.

A prominent feature of patients with schizophrenia is sleep disorder (4). From a biological rhythm perspective, the circadian rhythm—an endogenous, evolutionarily conserved timing system—generates approximately 24-hour cycles of physiological, behavioral, and metabolic oscillations through gene regulation (5). Influenced by environmental factors, habits, and disease, distinct activity patterns emerge, primarily categorized as morningness, eveningness, or intermediate chronotypes. This rhythm regulates human physiology and behavior and is closely linked to both physical and mental health. Emerging evidence suggests a correlation between eveningness chronotype—the subjective preference measured by instruments like MEQ-5—and increased aggression (6). While this study focuses on measurable chronotype phenotypes, broader circadian rhythm mechanisms may underlie these associations. Mechanistically, circadian rhythm disruptions may underlie this association, but the present study focuses on the measurable phenotype of chronotype. Among daily activities, sleep exhibits a highly structured pattern. Circadian disruptions can trigger sleep disturbances, particularly circadian rhythm sleep-wake disorders. Resulting imbalances in the sleep-wake cycle can lead to emotional dysregulation and, through the accumulation of negative emotions, increase the risk of aggression (7). Studies have shown that perceived social support, as a key psychological protective factor, can mitigate aggressive behavior by buffering the impact of stressful events and enhancing emotional regulation (8).

Current research suggests that circadian rhythms influence the mechanisms of aggressive behavior. However, most studies focus on the associations between sleep cycles, hormonal cycles, and conduct disorders, with limited research specifically linking chronotype to aggression. Furthermore, previous studies have found that aggression in patients with schizophrenia results from a combination of demographic, biogenetic, psychopathological, and psychosocial factors. Although considerable research explores relationships among circadian rhythms, sleep disorders, perceived social support, and aggression, few studies examine the interplay among these four elements, particularly in patients with schizophrenia. Therefore, this study aims to elucidate the relationships and underlying mechanisms linking circadian rhythms, sleep disorders, perceived social support, and aggressive behavior in patients with schizophrenia, providing a theoretical foundation for scientific management and intervention.

### Circadian rhythm and aggressive behavior

Circadian rhythm reflects an individual’s preference for sleep timing. Influenced by factors such as age, gender, and geographical latitude, the most distinct types of chronotypes are morningness and eveningness (9). Chronotype is strongly associated with sleep patterns; morning types typically maintain regular sleep schedules, demonstrating greater resilience to environmental disruptions and a greater tolerance for sleep disruptors (10). Conversely, evening types exhibit heightened vulnerability to disturbances in sleep-wake patterns. This irregularity constitutes a significant risk factor for mental disorders, with conditions such as sleep disorders and schizophrenia showing strong associations with the evening chronotype (11). The biological circadian system is governed by intricate regulatory mechanisms involving genes, hormones, and environmental cues (5). Key regulatory substances, such as cortisol, exhibit robust diurnal variation, typically peaking in the morning and declining throughout the day (12). Research has shown that individuals with relatively low daytime cortisol levels and elevated nocturnal levels demonstrate an increased risk for externalizing problem behaviors (12). Substantial evidence has linked sleep disturbances—particularly impaired quality or shortened duration—to externalizing behaviors like aggression, as shown in studies of both healthy populations and clinical groups (13). Notably, individuals with evening chronotypes exhibit a higher incidence of aggressive behavior.

Circadian dysregulation may affect brain circuits involved in emotion regulation, such as prefrontal-limbic system pathways, potentially increasing aggression risk (14). The hippocampus is especially critical; patients displaying aggression and antisocial personality disorder often show unilateral hippocampal atrophy and functional impairment (15). Notably, evening chronotypes exhibit specific associations with localized atrophy in the right hippocampal subiculum, which may contribute to their heightened aggression propensity through structural alterations.

Aggression in schizophrenia patients poses a substantial public health challenge, resulting in physical harm, perpetuating stigma, inflicting psychological distress, and escalating healthcare costs (16). It arises from multifactorial determinants including genetics, neurotransmitter systems, and neuroanatomical structure. Circadian rhythms may further modulate this aggression through hormonal/neurotransmitter regulation, as well as neurostructural plasticity. Building on this framework, we posit the following hypothesis:

H1: Circadian rhythm disruption is significantly associated with aggression in schizophrenia patients.

### The mediating role of sleep disturbances

Circadian rhythms are intrinsically linked to the regulation of sleep. The suprachiasmatic nucleus (SCN), the central circadian pacemaker, governs sleep timing, duration, architecture, and initiation, while also influencing wakefulness, alertness, cognitive function, and brain activity patterns (17). Research has indicated that a greater eveningness chronotype is linked to a higher risk of sleep disorders.

Sleep disturbances have been linked to increased impulsivity. Relevant studies have demonstrated that this is related to dysfunction of the medial prefrontal cortex (PFC) and the loss of top-down inhibitory control over the amygdala. This subsequently leads to excessive emotional responses to negative stimuli and an increase in emotional instability (18). According to the General Aggression Model, sleep disturbances may promote aggression through emotional, cognitive, and inhibitory control pathways (19). Additionally, the theory of hostile attribution bias suggests that poor sleep quality exacerbates the tendency to interpret ambiguous social cues as hostile, thereby triggering aggressive responses (20). Substantial evidence supported this connection (7, 21). Studies conducted by Stephen and Van et al. found a significantly higher aggressive behavior propensity in individuals with sleep disturbances, with poor sleep quality increasing aggression risk by 3.61-fold (22, 23). Given that sleep patterns are a primary circadian manifestation and sleep disturbances elevate aggression risk, we propose that sleep disturbances mediate the relationship between circadian rhythm dysfunction and aggression in patients with schizophrenia. Therefore, the impact of circadian disruption on aggression may operate partially through induced sleep pathology. Based on this rationale, we hypothesize:

H2: Sleep disturbances mediate the relationship between circadian rhythm dysfunction and aggressive behavior in schizophrenia patients.

Although circadian rhythms significantly influence aggressive behavior in schizophrenia patients, their impact varies substantially across individuals. Notably, even among patients sharing the same chronotype (e.g., evening type), the severity and likelihood of aggression differ. Consequently, identifying key moderating variables in this relationship is essential.

### The moderating effect of perceived social support

Social cognitive theory posits that social relationships profoundly shape cognition and behavior (24). Research has indicated that perceived social support effectively mitigates negative cognitions and associated maladaptive behaviors. Empirical work by Patti et al. further demonstrated that social support influences the manifestation of aggression by regulating cognitive processes (25). Simultaneously, the buffering model of social support suggested that perceived social support exerts beneficial effects regardless of stress levels (3), facilitating the reappraisal of stressful events. When excessive stress triggers negative emotions or behavioral tendencies (e.g., aggression), available social support buffers these adverse reactions.

Specifically in schizophrenia, circadian rhythms can elevate the risk of aggression via induced sleep disturbances. However, this risk manifests heterogeneously across patients, potentially moderated by their level of perceived social support. While sleep disturbances are a contributing factor to aggression, their severity is modulated by individual characteristics such as psychological resilience, stress tolerance, and adaptability (26). Crucially, support from family, friends, and social networks can enhance these capacities. Thus, perceived social support likely moderates the relationship between sleep disturbances and aggression: higher support may attenuate the adverse effects of sleep disturbances on aggressive behavior.

Recent studies corroborated the moderating role of perceived social support in the relationship between various factors and aggression (8, 27). For patients with schizophrenia who are experiencing significant stress and negative emotions, it plays a vital regulatory role in cognition and behavior. Based on this theoretical and empirical foundation, we propose:

H3: Perceived social support moderates the relationship between sleep disturbances and aggressive behavior in patients with schizophrenia.

In conclusion, this study employs a moderated mediation model to investigate the influence of circadian rhythm disruption on aggressive behavior in schizophrenia and its underlying mechanisms.

## Method

### Design and participants

This was a cross-sectional study based on self-report questionnaires administered at a single time point. From September 2020 to August 2021, patients with schizophrenia were recruited from 28 communities in Pengzhou, China (including Tianfu Middle Road Community, Tianfu Middle Road Small District, Guangming Community, Jinyang Community, Longtan Community, Linjiang Community, and Qingping Community) using a convenience sampling approach. Questionnaires were administered via the Questionnaire Star platform, a widely used online survey tool in China that allows researchers to design, distribute, and collect questionnaire data electronically. Recruitment consisted of psychiatrists and community nurses identifying potentially eligible patients from their registers based on inclusion and exclusion criteria. Subsequently, eligible patients and their families were contacted by community team members to explain the study objectives and procedures. For those who initially expressed interest, face-to-face meetings were arranged to obtain written informed consent. Community staff then assisted participants in accessing and completing the questionnaire through the Questionnaire Star platform on their personal smartphones or provided a tablet device for this purpose to ensure that participants with limited digital literacy could participate. The inclusion criteria for participants were as follows:

1. age ≥18 years (no upper age limit was set, as the study aimed to include a representative adult community sample);
2. diagnosis of schizophrenia confirmed by ≥2 psychiatrists according to the Diagnostic and Statistical Manual of Mental Disorders, Fifth Edition (DSM-5) criteria;
3. currently in clinical remission, as assessed by a psychiatrist, with a Positive and Negative Syndrome Scale (PANSS) total score <60, a commonly used cut-off indicating mild symptom severity and clinical stability in community-dwelling patients (28);
4. normal cognitive function, as determined by the treating psychiatrist’s assessment of the patient’s ability to comprehend and complete the study questionnaires; no formal cognitive screening scale was used;
5. voluntary provision of written informed consent.

Exclusion criteria were as follows:

1. other mental disorders, neurological diseases, brain developmental disorders, severe trauma, or significant physical illnesses (including but not limited to uncontrolled metabolic syndrome, hypertension, cardiovascular disease, severe diabetes, or other chronic conditions that could substantially affect sleep, chronotypes, or behavior);
2. a history of substance or alcohol dependence.

Regarding drug use, some participants are receiving stable antipsychotic medication treatment, using 1–3 types of psychotropic drugs as part of community therapy. The lack of systematic collection of drug details is considered a limitation of the potential confounding effects of psychotropic drugs on chronotype and aggression. This study was approved by the Ethics Committee of the Fourth People’s Hospital of Chengdu, with review number [2017], ethical examination number (16), and China Clinical Trial Registration Number ChiCTR1800015219, registration date March 15, 2018. Upon completion of the study, the researchers exported all survey data from the Questionnaire Star platform. A total of 818 participants were initially enrolled in the study. Prior to formal data collection, a pilot study was conducted to establish a minimum threshold for valid questionnaire completion. Under researcher supervision, 25 community-dwelling patients with stable schizophrenia completed the full set of questionnaires as instructed. The 10th percentile of completion time was 605 seconds. Given that the questionnaires covered multiple dimensions requiring deliberate responses (e.g., demographics, chronotype, aggression, sleep disturbance and social support), and based on the pilot data, responses completed in less than 600 seconds (10 minutes) were flagged as rapid and excluded. This conservative threshold, set just below the pilot P10 value, aimed to screen out inattentive responses while preserving valid data. After applying this criterion, 785 valid questionnaires were obtained, yielding an effective response rate of 95.97%.

*A priori* sample size calculation was performed using G*Power 3.1. For a linear multiple regression model with up to 6 predictors (including covariates), assuming a small to medium effect size (f² = 0.05), α = 0.05, and power = 0.80, the required sample size was 146. Our final sample of 785 exceeds this requirement, providing adequate statistical power.

### Measures

#### General demographic characteristics

General demographic characteristics reported by participants included gender, BMI, family residence, occupation, family monthly income (yuan), and marital status.

#### Chronotype assessment

In this study, the Chinese version of the Morningness-Eveningness Questionnaire-5 (MEQ-5) (29) was used to assess circadian preference (chronotype), reflecting a continuum from morningness to eveningness, rather than objective chronotype disruption. This scale is advantageous due to its simplicity and time efficiency. It employs a 4-point Likert-type scale, with total scores (ranging from 4 to 25) obtained by summing the responses to all five items. Lower scores correspond to an “eveningness” chronotype, while higher scores indicate a “morningness” chronotype. The Chinese MEQ-5 has demonstrated strong reliability and validity.

#### Aggressive behavior

Aggressive behavior was assessed by administering the revised Chinese version of the Modified Overt Aggression Scale (MOAS) (30, 31). This scale captures aggressive incidents over the past month, including verbal aggression, property aggression, self-aggression, and physical aggression toward others. Each subscale was rated on a 5-point Likert scale (0 to 4). The total MOAS score ranges from 0 to 16, summing the four subscales, with higher scores indicating more severe aggression. Cronbach’s α coefficients for the subscales ranged between 0.813 and 0.826.

#### Sleep disturbance

The Pittsburgh Sleep Quality Index (PSQI) was utilized to assess sleep quality (32). This scale consists of 18 items, including three open-ended questions, five multiple-choice questions, and 10 self-assessment questions. The total score is derived from seven factors, including sleep quality, sleep duration, the ability to fall asleep on time, sleep efficiency, sleep disturbances, hypnotic use, and daytime dysfunction. Scores on this scale range from 0 to 21, with higher scores indicating poorer sleep quality for the study subjects. On this scale, a total score exceeding 7 suggests the presence of sleep disturbances. The Cronbach’sα coefficient for the scale ranged from 0.703 to 0.778, indicating good internal consistency (33).

#### Social support

The Perceived Social Support Scale (PSSS), developed by Zimet et al., was administered in this study. The scale comprises three dimensions: family support, friend support, and support from significant others, comprising a total of 12 items. Responses were recorded using a 7-point Likert scale. The total score, which reflects an individual’s overall perceived social support, is calculated by summing the scores across all dimensions. Scores range from a minimum of 12 to a maximum of 84, with higher scores indicating greater perceived support and help from others (34).

### Statistical analysis

Data were analyzed using SPSS Statistics version 25.0 and the PROCESS macro (version 3.3). Prior to the analyses, all continuous variables were mean-centered. First, descriptive statistics and Spearman’s rank correlation analyses were conducted using SPSS for all variables. Next, PROCESS Model 4 was employed to examine the mediating effect of sleep disturbance. Subsequently, moderated mediation was tested using PROCESS Model 59. Finally, simple slope analysis was performed to examine whether the mediation effect of sleep disturbance (and chronotype) differed across different levels of the moderator variable. The following demographic variables were included as covariates in all statistical models: gender, body mass index (BMI categorized as<18.5,18.5-23.9,≥24kg/m^2^), family residence (rural/urban), occupation (categorized as unemployed, part-time/full-time employed, student, or other), monthly family income (categorized as ≤1000, 1001-3000, ≥3001 yuan), and marital status (single, married, widowed, divorced).Percentile bootstrap confidence intervals (CIs) were calculated based on 5,000 bootstrap samples.

### Common method bias

Data for this study were collected via self-report questionnaires, which may introduce common method bias (35). To mitigate this potential issue, procedural remedies (including participant anonymity and reverse-scoring of items) were implemented during data collection. Additionally, Harman’s single-factor test was conducted prior to analysis to assess standard method variance statistically. An exploratory factor analysis, which included all items measuring the four constructs (chronotype, sleep disturbance, perceived social support, and aggressive behavior), revealed six factors with eigenvalues greater than 1. The first factor accounted for 23.21% of the total variance, which is below the commonly accepted threshold of 40%. These results suggest that common method bias is unlikely to have substantially affected the findings of this study.

## Results

### Basic information and correlation analysis of the research subjects

This study included 785 community-dwelling patients with schizophrenia. The sample demographics were as follows: 414 (52.74%) male, 669 (85.22%) residing in rural areas, 555 (70.70%) unemployed, and 374 (47.64%) married. Regarding income distribution, the most significant proportion (n=402, 51.21%) reported a monthly income of ￥1001-3000. Based on BMI categories(<18.5kg/m²:underweight;18.5-23.9kg/m²:normal weight;≥24kg/m²: overweight/obese), 317 patients (40.38%) were overweight or obese. Marital status categories included single, married, widowed, divorced and others (After divorce or widowhood, having a new spouse). Aggressive behavior showed statistically significant differences across gender subgroups (χ² = 11.720, p = 0.001), with a higher percentage of females (18.06%) reporting aggressive incidents compared to males (9.66%). See **Table 1**.

**Table 1:** Aggressive behavior with different demographic characteristics.

Variable	n	Aggressive behavior		
		n(%)	χ2	*p*
Sex				
Male	414	40(9.66)	11.720	0.001
Female	371	67(18.06)		
BMI(kg/cm2)				
<18.5	39	7(17.95)	0.816	0.665
18.5∼23.9	329	46(13.98)		
≥24	317	54(17.03)		
Place of residence				
Rural area	669	97(16.95)	3.863	0.145
Town	86	9(10.47)		
City	30	1(3.33)		
Career				
No career	555	77(16.11)	5.858	0.053
Career	93	6(6.45)		
Other	137	24(17.52)		
Monthly income(yuan)				
<1000	290	46(15.86)	5.199	0.158
1001-3000	402	51(12.69)		
3001-5000	99	9(9.09)		
>5000	14	1(7.14)		
Marital status				
Unmarried	258	29(11.24)	5.62	0.229
Married	374	55(14.71)		
Divorced	127	18(14.17)		
Widowed	18	2(11.11)		
Other()	8	3(37.50)		

### Correlation analysis between variables

Partial correlation analyses among chronotype, sleep disturbances, aggressive behavior, and perceived social support, controlling for all demographic covariates (gender, BMI, residence, occupation, income, marital status), revealed significant associations. Sleep disturbances exhibited significant negative correlations with both chronotype and perceived social support, but a significant positive correlation with aggressive behavior. Chronotype demonstrated a significant positive correlation with perceived social support and a significant negative correlation with aggressive behavior. Additionally, perceived social support was significantly negatively correlated with aggressive behavior. Although the associations were statistically significant, their small effect sizes, all r < 0.2, suggest that the relationship is modest and may be influenced by other unmeasured variables. The mean MOAS score was low (0.32 ± 1.04). Only 13.63% of participants reported any aggressive incident (MOAS subscale score >0) in the past month, indicating a low base rate of overt aggression in this clinically stable community sample. Mainly because study entry criteria required patients to be clinically stable (PANSS total score <60), which may have selected for a less symptomatic population. Second, the community setting itself may represent a patient group with better disease control. And that patients may have social expectation bias or lack of insight when using self-rating instruments to measure aggressive behavior. Complete correlation coefficients and significance values are presented in **Table 2**.

**Table 2:** Descriptive statistics and correlation matrix of variables.

Variable	M	SD	1	2	3	4
1.Chronotype	17.36	2.45	1			
2.Sleep disturbance	4.00	2.92	-0.174^**^	1		
3.perceived social support	51.62	15.25	.101^**^	-0.109^**^	1	
4.aggressive behavior	0.32	1.04	-0.216^**^	.212^**^	-0.168^**^	1

**p<0.01.

### Test for moderated mediation effect

Using Hayes’ PROCESS macro (Model 4) in SPSS (36), we examined sleep disturbance as a mediator between chronotype and aggression. After controlling for demographic covariates (e.g., gender), chronotype significantly predicted sleep disturbance (a=-0.218, SE = 0.0347, *p* < 0.001). In the regression model including both predictors, chronotype retained a significant direct effect on aggression (c’ = -0.187, SE = 0.0347, *p* < 0.001), while sleep disturbance also significantly predicted aggression (b=0.178, SE = 0.0344, *p* < 0.001). Bias-corrected bootstrapping (5,000 samples) confirmed a significant indirect effect (ab=-0.032, BootSE=0.0122, 95% CI [-0.0594, -0.0117]), indicating that 14.22% of the total effect was mediated (ab/(ab + c’)). This suggests that disturbed sleep, although a statistically significant mediator, is not the only mechanism responsible for the total effect of chronotype on aggressive behavior. See **Table 3** for complete results.

**Table 3:** Mesomeric effect test of sleep disturbance between chronotype and aggressive behavior in schizophrenic patients.

Variable	Effect	BootSE	BootCI lower limit	BootCI upper limit	Effect proportion(%)
Total effect	-0.219	0.03	-0.286	-0.149	100
Direct effect	-0.187	0.03	-0.255	-0.119	85.39
Indirect effect	-0.032	0.01	-0.06	-0.01	14.61

In the second analytical phase, moderated mediation analysis was conducted using Model 59 of the SPSS PROCESS macro to test the moderating role of perceived social support. Three standardized regression equations were estimated simultaneously while controlling for demographic covariates (e.g., gender). Variance inflation factors (VIFs) remained ≤ 3.0 across models, indicating no concerns regarding multicollinearity.

**Figure 1:**
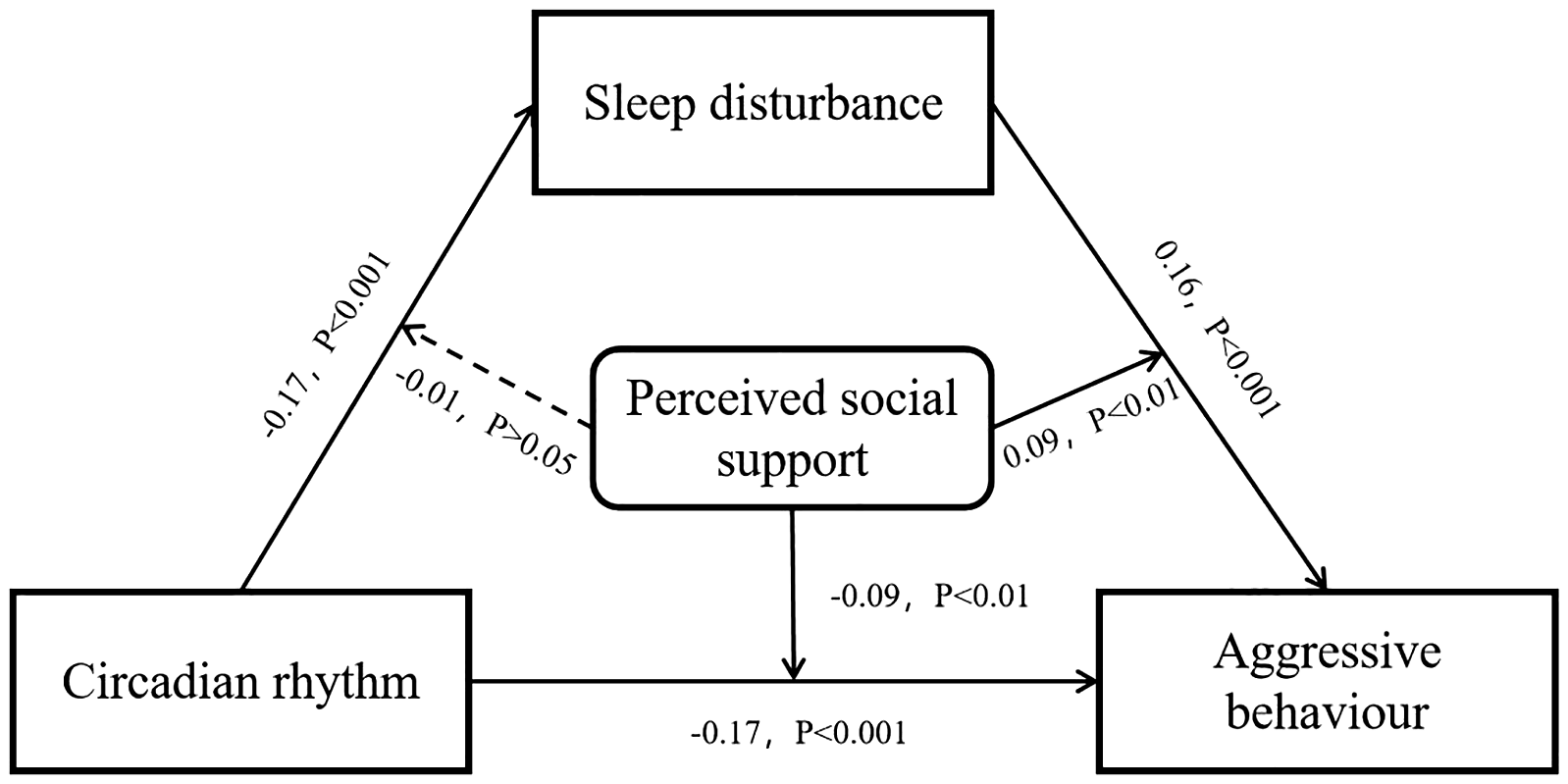
Moderating effect model.

As shown in **Table 4**, all conditions for moderated mediation were satisfied: (1) Chronotype significantly negatively predicted aggressive behavior (β = -0.19, *p* < 0.001); (2) Chronotype significantly negatively predicted sleep disturbance (β = -0.17, *p* < 0.001); and (3) Perceived social support demonstrated a significant main effect negatively predicting aggressive behavior (β = -0.13, *p* < 0.001), along with significant interaction effects were observed between social support and both chronotype (β = -0.09, *p* < 0.01) and sleep disturbance (β = 0.09, *p* < 0.01). These findings confirm that perceived social support moderates both the direct circadian rhythm-aggression pathway and the sleep disturbance-aggression pathway within the mediation framework. The complete moderated pathways are illustrated in **Figure 1**.

**Table 4:** Testing moderated mediation of chr-onotype on aggressive behavior.

Predictor	Model1			Model2			Model3		
	SE	β	T	SE	β	t	SE	β	t
Gender	0.07	0.19	2.80	0.08	0.49	0.70	0.07	0.18	2.72
Circadian rhythm	0.03	-0.19***	-5.38	0.35	-0.17***	-4.95	0.03	-0.17***	-4.98
perceived social support							0.03	-0.13***	-3.75
Sleep disturbance							0.03	0.16***	4.79
Circadian rhythm×perceived social support							0.04	-0.09**	3.03
Sleep disturbance×perceived social support							0.04	0.09**	-2.76
R^2^	0.30			0.17			0.35		
F	24.88***			12.45***			18.46***		

*p < 0.05; **p < 0.01; ***p < 0.001.

### Simple slopes analysis for moderating effects

To elucidate the interaction effects of perceived social support on chronotype and sleep disturbance, we conducted a simple slope analysis by dichotomizing perceived social support into high (M+SD) and low (M-SD) groups. We created a visualization of the simple effects.

**Figure 2:**
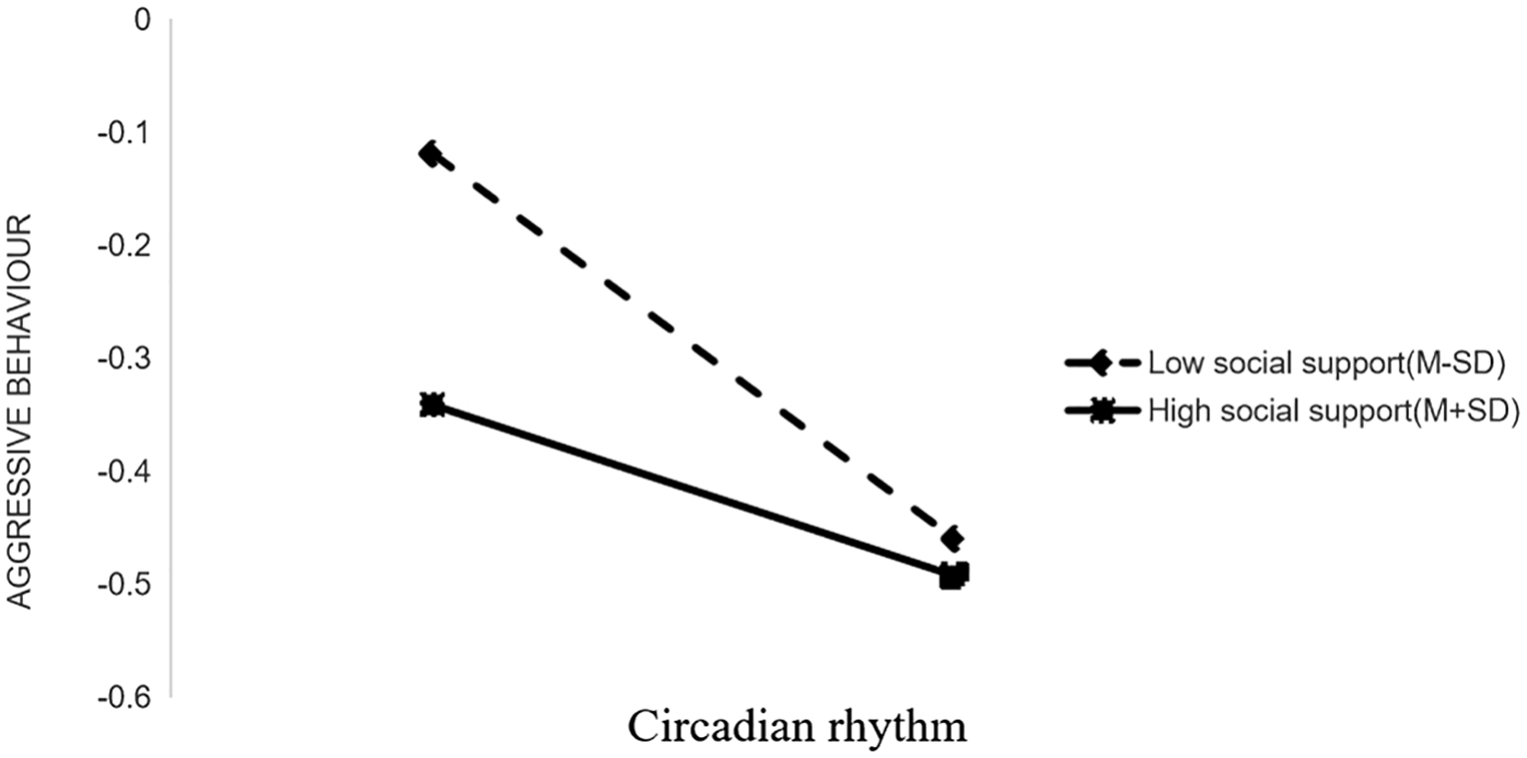
The moderating effect of perceived social support on the relationship between circadian rhythm and aggression behavior.

Simple slope analysis revealed distinct predictive patterns based on levels of perceived social support. For patients with schizophrenia in the low perceived social support group (M-SD), chronotype showed a significant negative prediction of aggressive behavior (Bsimple=-0.0265, t=-5.8482, *p* < 0.001). Conversely, this negative relationship was non-significant in the high perceived social support group (Bsimple=-0.756, t=-1.5947, *p*>0.05). These differential moderation effects are illustrated in **Figure 2**.

**Figure 3:**
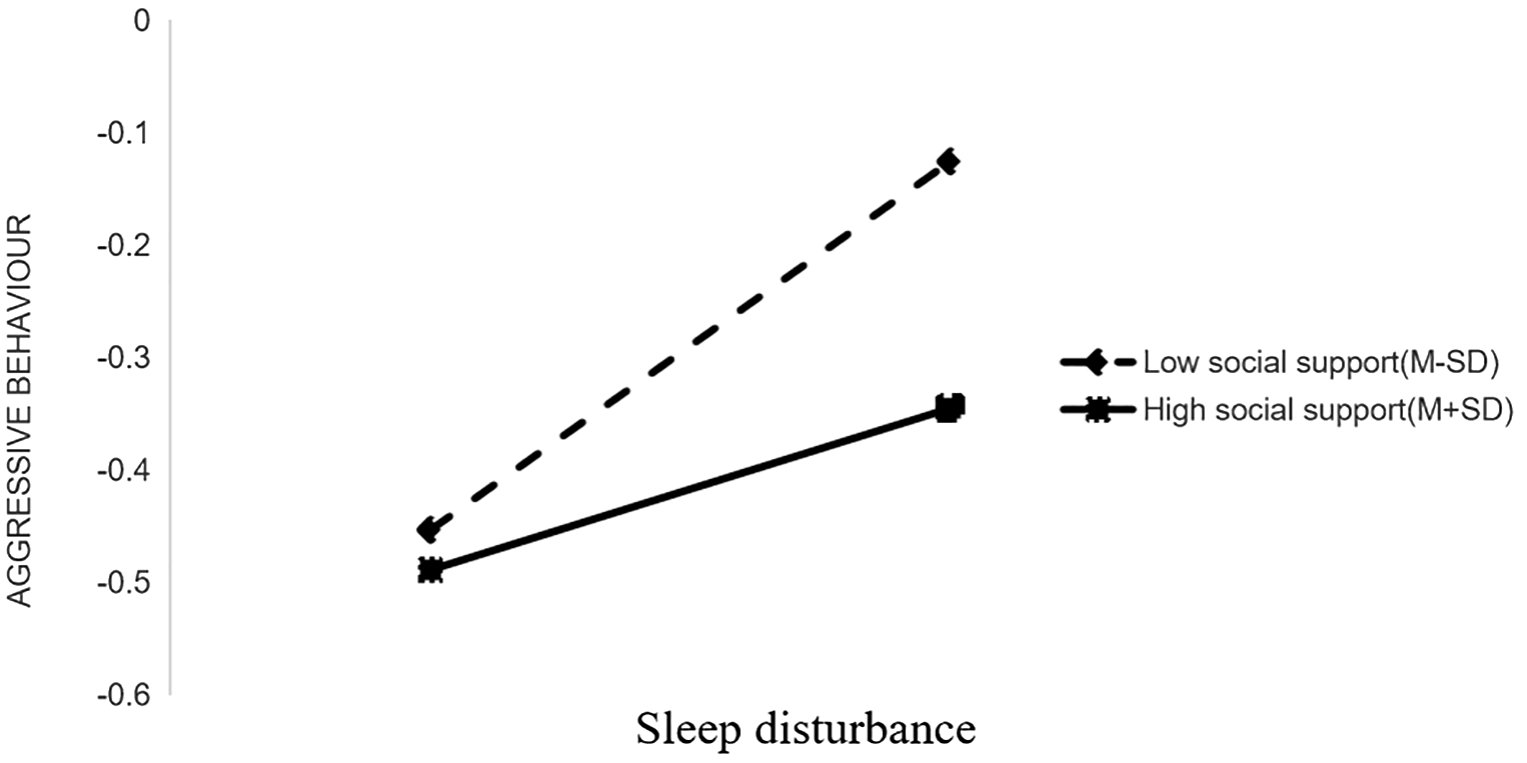
The moderating effect of perceived social support on the relationship between sleep disturbance and aggressive behavior.

In the sleep-disturbed chronotype pathway, sleep disorder significantly predicted aggressive behavior in schizophrenia patients with low scores (Bsimple=0.2559, t=5.4295, *p* < 0.001), whereas this prediction was not significant in patients with high scores (Bsimple=0.0720, t=1.4863, *p*>0.05) (see **Figure 3** for details).

Collectively, the findings indicated that the pathway linking chronotype disturbances to aggressive behavior via sleep disorder is mediated by perceived social support. Specifically, for schizophrenia patients with low perceived social support, the indirect effect of chronotype disturbances on aggressive behavior through sleep disorder was statistically significant (index=-0.0394, BootSE=0.0223, 95%CI [-0.0924, -0.0064]). In contrast, for patients with high perceived social support, this indirect effect was not statistically significant (index=-0.0128, BootSE=0.0102, 95% CI [-0.0359, 0.0041]).

## Discussion

This study investigated the complex interplay between circadian rhythm, sleep disturbances, perceived social support, and aggressive behavior in community-dwelling schizophrenia patients. The results revealed a nuanced mechanism: circadian rhythm was directly linked to aggressive behavior, with sleep disorders serving as a significant intermediary pathway. Furthermore, perceived social support moderates these effects, diminishing both (a) the direct circadian rhythm-aggression link and (b) the subsequent stage of the sleep disorder mediation pathway (sleep disorders to aggression). Crucially, circadian rhythm disturbances and sleep disorders exert a more pronounced impact on aggressive behavior in patients with low perceived social support compared to those with high support. These findings provide preliminary, theoretically relevant insights that can inform future longitudinal studies aimed at identifying at-risk individuals and designing targeted interventions to reduce aggressive behavior. It is important to interpret these findings in context. Although statistically significant, all correlation coefficients were below 0.22, indicating small effect sizes. Furthermore, the overall level of self-reported aggression was low, which is consistent with our sampling of clinically stable community patients. This limits the generalizability of the findings to more acute or hospitalized populations.

Previous research has linked circadian rhythm disturbances to aggressive behavior in individuals with schizophrenia. Our findings—characterized by relatively low overall aggression and a predominance of moderate morning-type chronotypes—further supported this inverse relationship between circadian integrity and aggression. Given the high prevalence of circadian disruption in schizophrenia and its established associations with exacerbated symptoms and impaired social functioning, these results underscore the clinical relevance of circadian regulation (37). Circadian rhythms are governed by a sophisticated biological system, with chronotype expression associated with genetic factors, sex differences, and environmental stimuli. Importantly, sleep attributes strongly connect to emotional processes (perception, regulation, expression), and behavioral changes correlate with circadian phase, suggesting direct involvement of circadian rhythms in neurobehavioral pathways for emotion and behavior regulation (38). In this study, patients identifying as morning types on the MEQ-5 reported better sleep quality, suggesting patterns consistent with more regulated sleep architecture in morning-oriented individuals. Stabilizing these processes mitigates aggression risk triggered by emotional liability or hormonal imbalances (39). Furthermore, emerging evidence suggests that polymorphisms in core clock genes may be associated with aggressive phenotypes and that a higher genetic risk load may increase susceptibility. Mechanistically, as shown by previous basic studies on epigenetic regulators such as Sirtuin 1 (SIRT1), circadian misalignment may affect aggression through dysregulation of transcriptional programs (40). However, because genetic or molecular markers were not measured in this study, these mechanisms remain speculative and need to be verified in future studies integrating biological assays.

This study examined the impact of circadian rhythm disturbances on sleep disorders and aggressive behavior in individuals with schizophrenia, utilizing a path model in which circadian rhythm serves as the predictor and sleep disorders act as the mediator. The results indicated that circadian dysregulation directly and negatively predicts aggressive behavior and also exerts an indirect effect via sleep disturbance mediation. Individuals with evening chronotypes demonstrated heightened vulnerability to sleep disturbances, This phenomenon was mainly related to subjective sleep delay. The results of this study support hypothesis H1 that there is a significant association between circadian preference and aggressive behavior. Compared with morning preference, evening preference reported higher levels of aggressive behavior. This finding is consistent with previous studies suggesting that circadian dysrhythmicity is a potential risk marker for aggressive behavior in schizophrenia (17). From a mechanistic perspective, patients with schizophrenia exhibit heightened susceptibility to this sleep pathology, which may involve multiple neurobiological alterations. Previous research indicates that dopaminergic dysfunction—a hallmark of schizophrenia—directly impacts sleep-wake regulation as a key circadian modulator (41). Concurrently, sleep deprivation may impair emotional regulation via prefrontal cortex dysfunction, thereby exacerbating aggressive behaviors (42, 43). The findings of this study provide indirect support for the aforementioned mechanisms. It should be emphasized, however, that this study relies solely on self-reported questionnaire data; these mechanistic interpretations require further validation through future research incorporating objective measurements. Our cross-sectional results are consistent with a model in which sleep disturbances partially explain the association between eveningness chronotype and aggression, establishing critical theoretical foundations and clinically actionable targets for the proposed causal cascade of circadian rhythm • sleep disorders • aggressive behavior in schizophrenia.

Crucially, this study identified perceived social support as a robust moderator in the relationship between the circadian rhythm and the aggression pathway. It notably reduced both: (a) the direct effect of circadian dysregulation on aggression, and (b) the latter segment of the mediated pathway (sleep disturbance → aggression). Mechanistically, high levels of perceived support mobilize multidimensional psychosocial resources—including emotional sustenance and instrumental aid—to mitigate stress- and negative affect-triggered behavioral dysregulation. This buffering effect substantially diminishes the adverse impact of circadian and sleep disruptions on aggression in individuals with schizophrenia (44). Perceived social support created a multi-layered defense system by enhancing psychological resilience. This system mitigates the emotional impact of sleep disorders, fosters a positive social atmosphere to alleviate negative emotions, and strengthens coping abilities during stress. Crucially, it interrupts the vicious cycle of stress accumulation → psychological collapse → externalizing aggression. In conclusion, as a fundamental bio-psycho-social protective factor, high perceived social support mitigates the risk of circadian rhythm disorders progressing to aggression through a tripartite pathway: emotion regulation, environmental optimization, and stress coping.

### Limitations and research perspectives

The overall framework of this study was cross-sectional, limiting the ability to establish causal relationships between variables. Future studies should employ longitudinal designs or intervention experiments to further verify causal mechanisms. Second, our sample was only from schizophrenia patients in Sichuan Province, limiting the generalizability of the results. Future studies should expand the sampling range to patients nationwide to improve representation. Third, the assessment of aggressive behavior relies entirely on self-assessment and may be affected by recall bias and underreporting, especially since individuals may not fully perceive or acknowledge the aggressiveness of their behavior. Fourth, the exclusion of patients with significant physical comorbidities (e.g., metabolic syndrome) may limit the representativeness of our sample regarding the broader community-dwelling schizophrenia population, which often presents with multimorbidity. Fifth, all key variables were assessed using self-rating instruments, which are susceptible to common method variance and biases such as social expectations and inaccurate recall. Future studies using objective measures (e.g., actigraphy for circadian rhythms and sleep, collateral reporting of aggressive behavior) would be more persuasive. Sixth, we did not systematically collect information on antipsychotic medications, which may influence circadian rhythms and aggression, thus representing a potential confounding factor. Additionally, data collection during the COVID-19 pandemic (2020-2021) may have influenced sleep patterns, social support, and aggression due to pandemic-related stressors, potentially affecting result generalizability. Finally, studies were not pre-registered, increasing the risk of analytical flexibility. Future studies should incorporate objective measures, use nationally representative samples, and consider other potential mediating and moderating variables to construct more comprehensive causal models.

## Conclusion

This study utilized a moderated mediation model to investigate the interactions among circadian rhythm, sleep disorders, perceived social support, and aggressive behavior in patients with schizophrenia, highlighting potential associations that could inform future longitudinal or intervention studies on community-based strategies.

## Data Availability

The original contributions presented in the study are included in the article/supplementary material. Further inquiries can be directed to the corresponding author.

## Glossary

PFC: prefrontal cortex
SCN: suprachiasmatic nucleus
PANSS: Positive and Negative Syndrome Scale
DSM-5: Diagnostic and Statistical Manual of Mental Disorders, Fifth Edition
MEQ-5: Morningness-Eveningness Questionnaire-5
MOASz: Explicit Offensive Behaviour Scale
PSQI: Pittsburgh Sleep Quality Index
PSSS: Perceived Social Support Scale
CI: confidence interval
DA: dopaminergic
5-HT: serotonergic
